# Correction: Strong light–matter interactions in hybrid polaritonic systems

**DOI:** 10.1039/d6cc90306a

**Published:** 2026-09-25

**Authors:** Ben Johns, Andrea Schirato, Federico Toffoletti, Tommaso Giovannini, Mirko Vanzan, Margherita Marsili, Giovanni Parolin, Giulia Dall’Osto, Ajay Kumar Poonia, Chiara Cappelli, Francesca Baletto, Stefano Corni, Elisabetta Collini, Margherita Maiuri, Nicolò Maccaferri

**Affiliations:** a Ultrafast Nanoscience Group, Department of Physics, Umeå University 90187 Umeå Sweden nicolo.maccaferri@umu.se; b Umeå Centre for Microbial Research 90187 Umeå Sweden; c Dipartimento di Fisica, Politecnico di Milano Piazza Leonardo da Vinci 32 20133 Milan Italy margherita.maiuri@polimi.it; d Department of Physics and Astronomy, Rice University 6100 Main Street Houston TX 77005 USA; e Department of Chemical Sciences, University of Padova via Marzolo 1 35131 Padova Italy stefano.corni@unipd.it elisabetta.collini@unipd.it; f Department of Physics, University of Rome Tor Vergata Rome Italy; g Department of Physics, University of Milan Via Celoria 16 20133 Milan Italy; h Dipartimento di Fisica e Astronomia “Augusto Righi”, University of Bologna Viale Berti Pichat 6/2 40127 Bologna Italy; i Elettra Sincrotrone Trieste SS 14 Km 163.5 in AREA Science Park Basovizza Trieste Italy; j Wallenberg Initiative Materials Science for Sustainability, Department of Physics, Umeå University 90187 Umeå Sweden; k Scuola Normale Superiore Piazza dei Cavalieri 7 I-56126 Pisa Italy; l Istituto Nanoscienze – CNR, S3 Via G. Campi 213/A 41125 Modena Italy

## Abstract

Correction for ‘Strong light–matter interactions in hybrid polaritonic systems’ by Ben Johns *et al.*, *Chem. Commun.*, 2026, https://doi.org/10.1039/d6cc02672a.

The authors regret that three of the corresponding authors’ email addresses were omitted in the original article. The correct author details are provided here.

The Royal Society of Chemistry apologises for these errors and any consequent inconvenience to authors and readers.

